# Genome-wide insights of Ethiopian indigenous sheep populations reveal the population structure related to tail morphology and phylogeography

**DOI:** 10.1007/s13258-020-00984-y

**Published:** 2020-08-16

**Authors:** Agraw Amane, Gurja Belay, Yao Nasser, Martina Kyalo, Tadelle Dessie, Adebabay Kebede, Tesfaye Getachew, Jean-Baka Domelevo Entfellner, Zewdu Edea, Olivier Hanotte, Getinet Mekuriaw Tarekegn

**Affiliations:** 1 Department of Microbial, Cellular and Molecular Biology, Addis Ababa University, Addis Ababa, Ethiopia; 2 Andassa Livestock Research Center, Amhara Regional Agricultural Research Institute, Bahir Dar, Ethiopia; 3 Biosciences Eastern and Central Africa-International Livestock Research Institute (BecA-ILRI) Hub, Nairobi, Kenya; 4 LiveGene Program, International Livestock Research Institute, Addis Ababa, Ethiopia; 5 International Center for Agricultural Research in the Dry Areas, Addis Ababa, Ethiopia; 6 Department of Animal Science, Chungbuk National University, Cheongju, South Korea; 7 Department of Animal Breeding and Genetics, Swedish University of Agricultural Science (SLU), Uppsala, Sweden; 8 Department of Animal Production and Technology, School of Animal Sciences and Veterinary Medicine, Bahir Dar University, Bahir Dar, Ethiopia; 9 School of Life Sciences, University of Nottingham, University Park, Nottingham, NG7 2RD UK

**Keywords:** Fat-tail, Genetic diversity, Ovine 50 K SNP, Population structure

## Abstract

**Background:**

Ethiopian sheep living in different climatic zones and having contrasting morphologies are a most promising subject of molecular-genetic research. Elucidating their genetic diversity and genetic structure is critical for designing appropriate breeding and conservation strategies.

**Objective:**

The study was aimed to investigate genome-wide genetic diversity and population structure of eight Ethiopian sheep populations.

**Methods:**

A total of 115 blood samples were collected from four Ethiopian sheep populations that include Washera, Farta and Wollo (short fat-tailed) and Horro (long fat-tailed). DNA was extracted using Quick-DNA™ Miniprep plus kit. All DNA samples were genotyped using Ovine 50 K SNP BeadChip. To infer genetic relationships of Ethiopian sheep at national, continental and global levels, genotype data on four Ethiopian sheep (Adilo, Arsi-Bale, Menz and Black Head Somali) and sheep from east, north, and south Africa, Middle East and Asia were included in the study as reference.

**Results:**

Mean genetic diversity of Ethiopian sheep populations ranged from 0.352 ± 0.14 for Horro to 0.379 ± 0.14 for Arsi-Bale sheep. Population structure and principal component analyses of the eight Ethiopian indigenous sheep revealed four distinct genetic cluster groups according to their tail phenotype and geographical distribution. The short fat-tailed sheep did not represent one genetic cluster group. Ethiopian fat-rump sheep share a common genetic background with the Kenyan fat-tailed sheep.

**Conclusion:**

The results of the present study revealed the principal component and population structure follows a clear pattern of tail morphology and phylogeography. There is clear signature of admixture among the study Ethiopian sheep populations

**Electronic supplementary material:**

The online version of this article (10.1007/s13258-020-00984-y) contains supplementary material, which is available to authorized users.

## Introduction

Given its proximity to the Arabian Peninsula, Ethiopia is considered as a corridor for the introduction of livestock species including sheep to the African continent (Hanotte et al. 2002; Muigai and Hanotte 2013). Sheep and their products play a critical role in the livelihood of millions of farmers and pastoral communities in Ethiopia and are important for the national economy (Assefa et al. 2015). Ethiopia possesses highly diversified indigenous sheep populations adapted to highly diverse agro-ecologies and the populations are maintained by difference ethnic communities (Haile et al. 2002; Gizaw et al. 2008). There are about 29.33 million heads of sheep which are phenotypically identified into 14 populations (Gizaw et al. 2008; Leta and Mesele 2014). However, the local sheep populations with very low productivity dominate smallholder production systems, which are mainly confounded by lack of effective long-term sheep genetic improvement, multiplication and effective delivery systems, environmental as well as socio-economic factors (Tadele 2010; Gizaw et al. 2013). On the other hand, the demand for sheep and their products has increased from time to time due to a growing human population and urbanization. Therefore, it is an urgent need to improve their productivity in order to raise smallholder farmers’ incomes, to meet the demand of the growing human population as well as the national economy (FAO 2012).

The local sheep populations in Ethiopia are mainly named after the geographic location or ethnic group, based on phenotypic characteristics or agro-ecology (Solomon et al. 2011). This might have led to group genetically similar populations into phenotypically distinct groups. The previous studies indicated that Ethiopian indigenous sheep were clustered together based on their geographic distribution and tail phenotypes (Gizaw et al. 2007; Edea et al. 2017; Ahbara et al. 2019). Characterizing genetic diversity and understanding of population structure is a key aspect of developing sustainable breed improvement strategies (Groeneveld et al. 2010) and understanding adaptation to extreme environments (Ai et al. 2014). Molecular characterization were done on genetic diversity and population structure of Ethiopian sheep populations using microsatellite markers (Gizaw et al. 2007; Hellen 2015). In a recent study, Edea et al (2017) and Ahbara et al (2019) evaluated population structure of Ethiopian indigenous sheep populations (Kefis, Adane, Arabo, Gafera, Molale, Bonga, Gesses, Kido, Doyogena, ShubiGemo, Loya, Adilo, Menz, Arsi-Bale, Horro and Black Head Somali) and revealed high level of admixture among the populations using high and medium density SNP chip panel, respectively. Recently developed genome-wide ovine SNP array has provided a tool for investigating genetic diversity at high resolution, inferring population history, and mapping genomic regions subject to selection and adaptation (Kijas et al. 2009; Yang et al. 2016; Zhao et al. 2017).

However, there are still indigenous sheep populations in Ethiopia yet to be evaluated at genome-wide. These sheep populations include Farta, Wollo, Washera and Horro sheep which are adapted to diverse agro-ecologies and with different tail phenotype. Therefore, in this study we aimed to investigate genome-wide genetic diversity and population structure of the indigenous sheep populations in Ethiopian.

## Material and methods

### Sheep populations, sampling and SNP genotyping

Blood samples were collected from four Ethiopian sheep populations (n = 115) adapted to diverse agro-ecological environments. Detail description of the populations is summarized in Table 1. Farta (n = 26) and Wollo (n = 28) sheep are short fat-tailed coarse-wool sheep and adapted to sub-alpine environments (2000–3200 m a.s.l); Washera (n = 31) sheep is short fat-tailed hairy sheep and mainly inhabits wet, warmer mid-highlands (1600–2497 m a.s.l); Horro (n = 30) sheep is long fat-tailed hairy sheep and predominant in mid-to high-altitude environments (1400–2000 m a.s.l) (Gizaw et al. 2008). Geographic positioning system (GPS) coordinates were recorded for each sheep population and geographical distribution map was developed based on their GPS coordinates (Supplementary Fig. 1).

**Table 1:** Distinguishing physical features and agro-ecology of Ethiopian sheep populations

Physical features and their ecology	Population							
	Washera	Farta	Horro	Wollo	BHS	Arsi-Bale	Menz	Adilo
Fiber type	Short-haired	Coarse wool	Short-haired	Coarse wool	Short-haired	Hairy fiber	Coarse wool	Hairy fiber
Tail phenotype	Short fat-tail	Short fat-tail	Long fat-tail	Short fat-tail	Short fat-rump	Long fat-tail	Short fat-tail	Long fat-tail
Coat color	Brown, white, white and brown	White, brown, black	Brown, fawn	Black, white or brown	White body, black head and neck	Brown, red, black, gray, white	Black, Brown, white	Brown, white, gray
Horn	Polled	Male short horned, most females polled	Polled	Male short horned, most females polled	Polled	Males and most females are horned	Horned	Male short horned, most females polled
Altitude (m)	1600–2497	2000–3142	1400–2000	2000–3200	500–1500	2492–2810	2500–3000	1618–2043
Agro-ecology	Wet, warmer mid- highlands	Sub-alpine	Wet highlands	Sub-alpine	Arid lowland	Wet highland	Sub-alpine	Wet highland
Use	Meat	Meat & wool	Meat	Meat &wool	Meat	Meat	Meat &wool	Meat
Management	Mixed crop-livestock	Mixed crop-livestock	Mixed crop-livestock	Mixed crop-livestock	Pastoral/agro-pastoral	Mixed crop-livestock	Sheep-barley	Mixed crop-livestock
Community	Amhara and Agew	Amhara	Oromo	Amhara	Oromo/Somali	Oromo	Amhara	Wolaita/Hadiya

*BHS* black head Somali

Source: Gizaw et al. (2008); Edea et al. (2017)

The samples were collected from different households in different villages using 5 ml of vacutainer tube with 1 ml EDTA as anti-coagulant. A maximum number of 1–3 distinct breeding adult animals were sampled per household based on flock owners’ information and typical phenotypic characteristics that allow to avoid sampling of related animals. Genomic DNA was extracted using Quick-DNA™ Miniprep plus kit following the procedures of Biological Fluid and Tissue protocol (https://WWW.zymoresearch.com/m/D4068). All 115 genomic DNA samples were genotyped using Ovine 50 K SNP BeadChip (Illumina, San Diego, CA, USA) by GeneSeek/Neogen (Lincoln, NE, USA).

To infer genetic relationships of Ethiopian sheep populations at national level, Ovine 600 K SNP BeadChip genotype data of 44 animals representing four extra Ethiopian sheep populations (Adilo = 11; Arsi-Bale = 8; Blackhead Somali = 13; Menz = 12) were included in the study. Detail description of the extra four Ethiopian sheep populations is summarized in Table 1. The genotype data (600 K) were obtained from NRSP-8 Community File Sharing Platform (https://www.animalgenome.org/repository/pub/KORE2017.1122/).Ovine 50 K SNP BeadChip genotype data of 105 animals representing six breeds from east (Kenya: Red Maasai), north (Egyptian Barki; Moroccan sheep) and south (South Africa: Namaqua Afrikaner) Africa, Middle East (Iran: Afshari) and south-west Asia (India: Garole) were included in the study to investigate the genetic relationships in greater detail and infer population structure of Ethiopian sheep populations at continental and global levels. The genotype data (50 K) were obtained from Sheep HapMap and Animal Resources dataset (https://www.sheephapmap.org/hapmap.php). Therefore, a total of 14 sheep populations, including extra four Ethiopian sheep, are included in the study as reference.

### Quality control and genetic diversity analyses

Genotypic data for 600 K and 50 K platforms were merged and common autosomal SNP markers (42,227) were obtained. These common autosomal SNPs with call rate less than 90% and minor allele frequency (MAF) less than 0.05 were filtered out using PLINK v1.9 (Purcell et al. 2007) and leaving 37,771 autosomal SNPs. Using the same software, this generated dataset were further subjected to linkage disequilibrium (LD) pruning to avoid the possible influence of clusters of SNPs on population relationships and structure analyses (Yuan et al. 2017). Following the LD pruning, 30,292 autosomal SNPs were retained for population structure analyses.

To evaluate the levels of within-population genetic diversity, the expected (*H*_E_) and observed (*H*o) heterozygosity and inbreeding coefficient (*F*) were estimated for each population using Arlequin v.3.5.2 (Excoffier and Lischer 2010). To partition genetic variation among groups, among population within groups and individuals within population, analysis of molecular variance (AMOVA) was performed following 10,000 permutations in Arlequin v.3.5.2 (Weir and Cockrham 1984). The analysis was done for the 14 global sheep populations grouped into major clusters based their geographical origin and community the populations are kept. This include East African (Ethiopia and Kenya), Arab region (Egypt, Morocco and Iran), India (Indian Garole sheep) and South African (Namaqua Afrikaner sheep). We further regrouped Ethiopian sheep populations separately according to their tail phenotype (Short fat-tailed: Wollo, Farta, Washera and Menz; Long fat-tailed: Arsi-Bale, Adilo, and Horro; Fat rumped: Blackhead Somali) and geographical location (Northern: Washera, Farta, Wollo and Menz; Southern: Adilo, Arsi-Bale and Horro; Eastern: Blackhead Somali).

### Genetic population structure analyses

Principal components analysis (PCA) was performed using PLINK v1.9 (Purcell et al. 2007) to investigate the genetic structure and relationships among the studied populations based on genetic correlations between individuals. A graphical display of the first two principal components (PC1 and PC2) was generated using gglot2 package provided by R (Wickham 2009). Population structure analyses carried out in STRUCTURE v.2.3.4 (Pritchard et al. 2000) was used to investigate underlying genetic structure and estimate the proportion of shared genome ancestry between the studied populations. The STRUCTURE output was further analyzed in STRUCTURE HARVESTER (Earl 2012) to determine the optimal number of ancestral genomes (*K*) and proportions of genome ancestry shared among the studied populations using ΔK method (Evanno et al. 2005). To further evaluate the within and between Ethiopian and global sheep populations relationships, we used pairwise genetic differentiation estimates to construct the neighbor-network tree using SplitsTree4v.4.6 (Huson and Bryant 2005).

## Results

### Genetic diversity

**Fig. 1:**
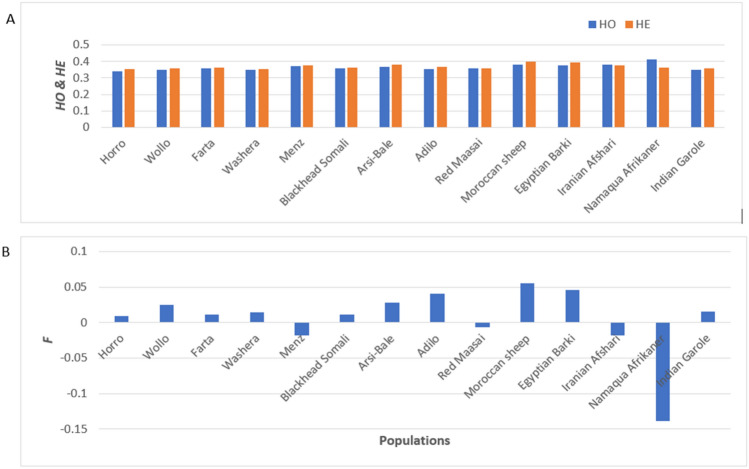
Distribution of the genetic diversity indices within each population. **a** observed heterozygosity (*H*o); expected heterozygosity (*H*_E_) and **b** the inbreeding coefficient (*F*)

The mean values of *H*_E_, *H*o and *F*, as measures of within-population genetic diversity, for 14 sheep populations are shown in Fig. 1 and supplementary Table S1. The lowest values of *H*_E_ and *H*o were observed in Horro sheep while the highest values were observed in Moroccan, Egyptian Barki, Arsi-Bale and Menz sheep populations, respectively. The highest and lowest inbreeding coefficient (*F*) was observed in Moroccan and Namaqua Afrikaner sheep populations, respectively. Analysis of molecular variance for the 14 global populations grouped according to geographical regions revealed that 9.67% (*P* < 0.0001) of the variance was among groups (East Africa; Arab region; India and South Africa) (Table 2). When an analysis was performed for Ethiopian sheep populations grouped based on their tail phenotype (Table 1), 3.14% (*P* < 0.001) of the variance was among groups (Table 2). We further ran AMOVA by grouping the Ethiopian sheep populations according to their geographical distribution (Table 1). Results indicated that 4.67% (*P* < 0.001) of the variation was among groups, 3.40% among populations within groups and 91.93% within populations (Table 2).

**Table 2:** Analysis of molecular variance of the studied sheep populations from analysis of 37,771 SNPs

Grouping	Source of variation	DF	Variance components	Percentage of variation
Geographical region (Inter-regional)	Among groups	3	727.082 Va	9.67
	Among population within groups	**301**	332.156 Vb	4.42
	Within populations	305	6462.026 Vc	85.92
	Total	609	7521.264	100
Tail phenotype	Among groups	2	213.379 Va	3.14
	Among population within groups	**197**	174.803 Vb	2.58
	Within populations	200	6397.488 Vd	94.28
	Total	399	6785.669	100
Geographical region (Inter-local)	Among groups	2	721.083 Va	4.67
	Among population within groups	**197**	255.405 Vb	3.40
	Within populations	200	6361.020 Vd	91.93
	Total	399	7337.508	100

Bold values indicate degree of freedom within population

*DF* degree of freedom, *Va, Vb, Vc, Vd* explain null distributions of the variance; P < 0.0001; components in the respective sources of variance (Weir and Cockrham 1984)

### Principal component analyses

**Fig. 2:**
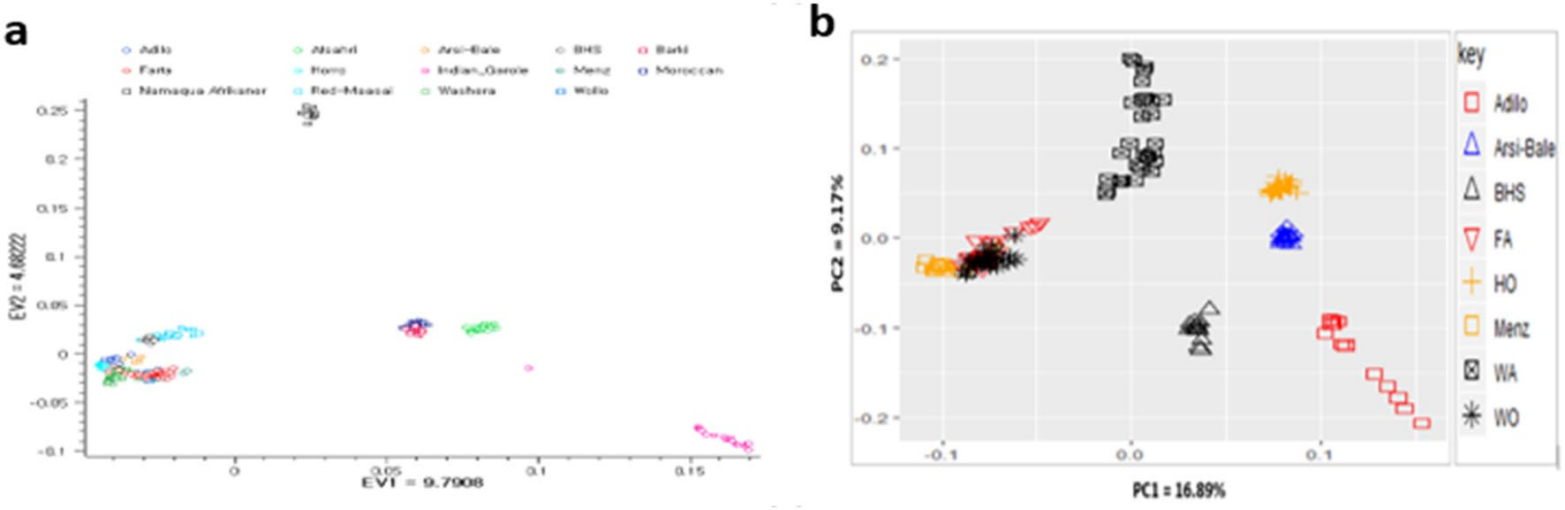
Clustering of individual animals in the studied sheep populations **a** in a global and **b** Ethiopian indigenous sheep populations datasets

**Fig. 3:**
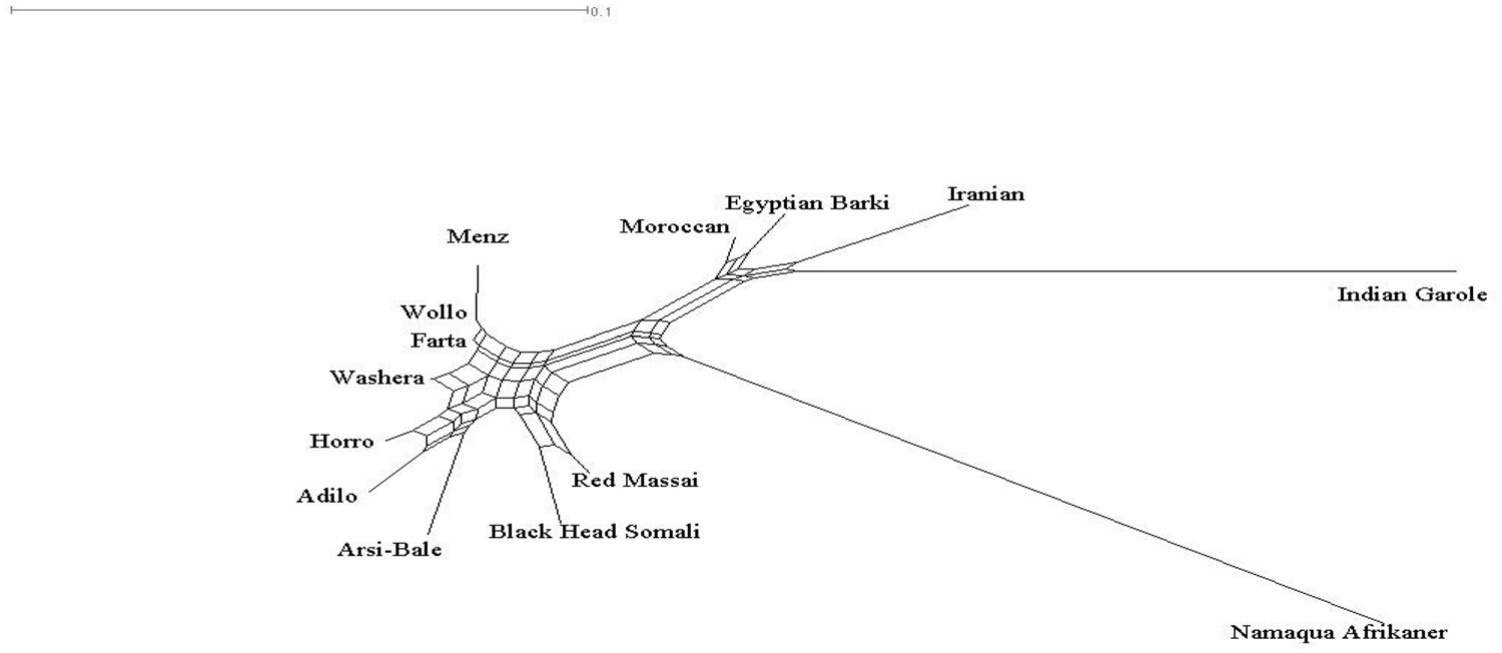
Neighbor network constructed in a global geographic context

**Fig. 4:**
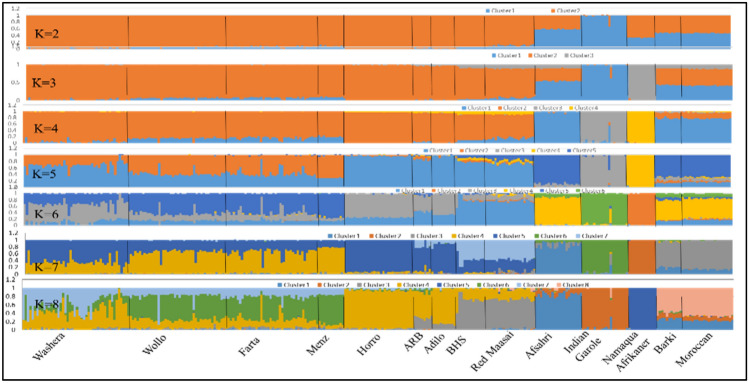
Population structure analyses of the studied populations in a global context. The eight genetic clusters are designated (Cluster 1) ~ (Cluster 8), respectively

The principal component analysis (PCA) plot incorporating the global populations indicated that the 14 sheep populations are differentiated based on regions (east Africa: Kenya and Ethiopia; North Africa: Moroccan and Barki sheep populations; South Africa: Namaqua Afrikaner; Middle East: Afshari; India: Garole) (Fig. 2a). Moreover, the east Africa sheep populations differentiated based on their tail morphology. PC1 separates east Africa populations from South Africa, North Africa, Middle East and India sheep populations. Ethiopian (except BHS) and India populations are separated from east Africa, north Africa, South Africa and Middle East sheep populations by PC2. Among east Africa populations, Ethiopian fat-rumped sheep, BHS, and Kenyan fat-tailed sheep, Red Maasai, cluster together which is well supported by phylogenetic and structure analysis results (Figs. 3, 4, respectively).

To illustrate relationships within individuals and among Ethiopian sheep populations, PCA was performed (Fig. 2b). Principal components 1 and 2 accounted for 16.89% and 9.17% of the total variation, respectively and clustered the eight sheep populations according to their tail phenotypes and geographical distribution: long fat-tailed (Arsi-Bale, Horro, Adilo), short fat-tailed (Washera, Farta, Wollo, Menz), and fat-rumped (Black Head Somali). PC1 separate short fat-tailed sheep (FA: Farta, WO: Wollo, and Menz) from the rest of sheep populations including most individual animals of the other short fat-tailed sheep (WA: Washera). PC2 separate the short fat-tailed sheep (WA:Washera), the long fat-tailed sheep (Horro), few individuals of Farta and Arsi-Bale sheep from the rest of sheep populations.

### Phylogenetic network analysis

A Neighbor-Net network constructed from pairwise comparison cluster the global dataset (14 sheep populations) according to their geographic region and tail phenotype as indicated in Fig. 3. The line in the phylogenetic network refers to population subdivision and the nodes refers to subpopulations. Among Ethiopian sheep, the short fat-tailed sheep (Farta, Wollo and Washera) were closely clustered. The two highland and long fat-tailed Ethiopian sheep (Horro and Adilo) were closely clustered. The Ethiopian fat-rumped (BHS) and the Kenyan fat- tailed (Red Maasai) sheep as well as the two North Africa sheep (Egyptian Barki and Moroccan sheep) were closely clustered. Long branches were noted for Namaqua Afrikaner (fat-tailed), Indian Garole (thin-tailed), and Iranian Afshari (thin-tailed) sheep possibly due to proximity in geographical region and their tail variation which could be associated to adaptation. The findings were further supported by global principal component and population STRUCTURE analyses (Figs. 2a, 4, respectively).

The close clustering of East African sheep populations and distinct separation from their Northern counterparts was well demonstrated by our phylogenetic and global PCA analysis results (Figs. 3, 2a, respectively).

### Genetic population structure analyses

**Fig. 5:**
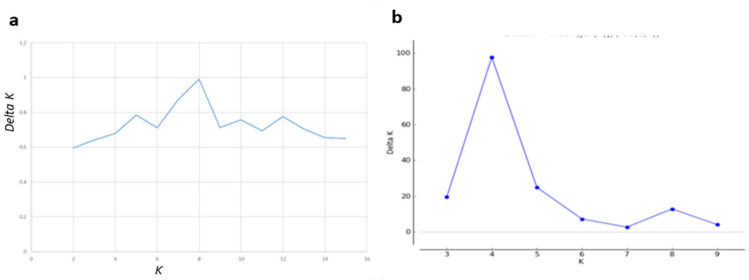
Graph of cross validation (CV) error generated for: **a** global and **b** Ethiopian indigenous sheep populations datasets

Genetic population structure analysis on the global dataset (14 sheep populations) was carried out using hypothetical ancestral clusters (*K*) ranging from 2 to 15 and cluster the studied populations according to their geographic region and tail phenotype (Fig. 4). The highest ∆*K* value registered at *K* = 8 suggesting this to be the most optimal number of clusters explaining the variation in the dataset (Fig. 5a). The proportion of each ancestral cluster in each population at *K* = 8 is shown in Fig. 4 and Supplementary Table S2. Indian Garole and Namaqua Afrikaner sheep separated from the rest of sheep populations at *K* = 2 and *K* = 3, respectively. East Africa (Ethiopia and Kenya) sheep populations separated from the rest of the sheep populations at *K* = 4.

At optimal *K* (*K* = 8), Ethiopian (Blackhead Somali) and Kenyan (Red Maasai) sheep share up to 94% a single common genetic background (Cluster 3). The Asian thin-tailed sheep, Indian Garole, formed an independent cluster (Cluster 2) with high proportion (~ 95%). The South Africa fat-tailed sheep, Namaqua Afrikaner, formed a clear separate cluster (Cluster 5) with the highest proportion (~ 100%) from the rest of the populations. The two North African representatives shared one genetic background (Cluster 8), and both shared 22–26% common genetic background with Afshari of Iranian sheep (Cluster 1). The Asian thin-tailed sheep showed distinct clustering from east Africa and South African fat-tailed and from east Africa (Ethiopia) fat-rumped sheep.

**Fig. 6:**
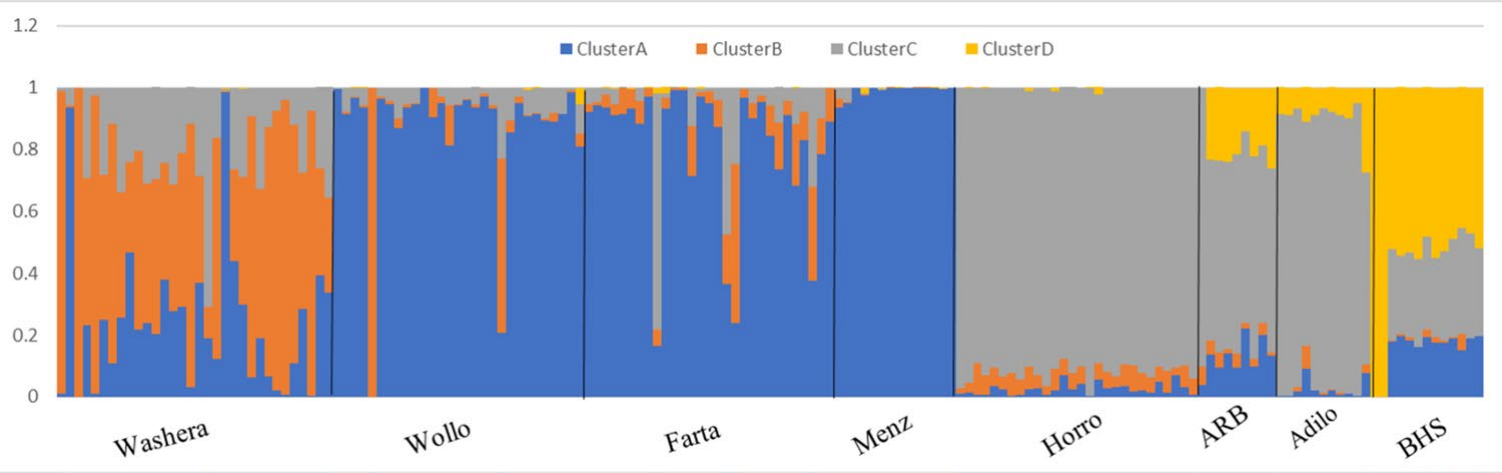
Population structure analyses of Ethiopian sheep

Three to nine hypothetical ancestral clusters (*K*) were tested with structure on the Ethiopian dataset (national dataset). The highest ∆*K* value suggested *K* = 4 as most optimal number of ancestral clusters present in the national dataset (Fig. 5b). The proportion of each ancestral cluster in each population at *K* = 4 is shown in Fig. 6 and Supplementary Table S3. Menz (highest proportion ~ 100%), Wollo, Farta and few individuals of Washera sheep share one genetic cluster group (Cluster A). Cluster B observed in Washera (proportion ~ 55%) and few individuals of Wollo, and Farta sheep. Horro, Arsi-Bale (ARB) and Adilo sheep form Cluster C whereas Cluster D observed in Blackhead Somali (BHS) and some individuals of Arsi-Bale (ARB) and Adilo sheep. Ethiopian short fat-tailed sheep (Washera, Wollo, Farta, and Menz) did not represent one genetic cluster group rather they form two different genetic cluster groups (Cluster A and B) which is supported by admixture analysis for other Ethiopian short fat-tailed sheep populations (Ahbara et al. 2019). Farta, Wollo and Menz sheep formed one genetic cluster group (Cluster A) while Washera and few individuals of Farta and Wollo sheep formed another genetic cluster group (Cluster B). Close clustering was observed among Ethiopian long fat-tailed sheep (Horro, Adilo, and Arsi-Bale) (Cluster C). The findings are further supported by national principal component and global sheep genetic population structure analyses results (Figs. 2b, 4, respectively).

## Discussion

The genotype data generated by Ovine 50 K SNP BeadChip were used to investigate genetic diversity and population structure of Ethiopian indigenous sheep populations. To assess the genetic diversity and relationships in greater detail and infer population structure of Ethiopian sheep populations at the continental and global levels, populations from other regions of the African continent and the world were included. The findings revealed that the estimated within Ethiopian sheep population diversity is in line with previous studies on indigenous sheep populations in Ethiopia using microsatellite markers and SNP chip (Gizaw et al. 2008; Edea et al. 2017; Ahbara et al. 2019). The presence of high genetic diversity within-population is congruent with the high variability observed in phenotypic characters, particularly in coat color within the sub-alpine sheep populations (Gizaw et al. 2008). High degree of genetic diversity within-population is a characteristic of large traditional populations that have not been subjected to strong selection indicating the need to conserve such traditional populations (Lauvergne et al. 2000), and thus can be exploited through implementing appropriate breeding strategies.

Ethiopian indigenous sheep showed slightly lower levels of genetic diversity (*H*_E_ = 0.366) than the presumed ancestral populations in the Middle East (Afshari; *H*_E_ = 0.376) and North Africa (*H*_E_ = 0.401). Populations found near to domestication center are expected to retain higher allelic diversity than those that migrated farther away (Peter et al. 2007). The higher level of diversity estimates in North Africa as compared to east Africa populations can be further explained by the fact that North Africa populations reflect a high degree of admixture between fat-tailed and thin-tailed sheep as demonstrated in the STRUCTURE analyses result (Fig. 4 and Supplementary Table S2). Given its proximity to the Near East and Mediterranean Sea, North Africa served as a gateway for early livestock introduction to the African continent and is considered as a secondary hotspot of genetic variation (Gautier 2002).

Although they are defined by the same tail phenotype, the four Ethiopian short fat-tailed sheep (Wollo, Menz, Farta and Washera) did not cluster together. Adaptation to different eco-climates that can impede gene flow, may have shaped this genetic sub-structuring (Madrigal et al. 2001; Gizaw et al. 2007). The close clustering of the three short fat-tailed sheep (Wollo, Farta and Menz) is consistent with adaptation to similar eco-climates (sub-alpine), similar in tail phenotype and they are maintained by the same ethnic group or community which may act as barriers to gene flow that shape population genetic structure (Madrigal et al. 2001). The distinct clustering of the warmer mid-highland short fat-tailed sheep (Washera) from the three sub-alpine short fat-tailed sheep (Wollo, Farta and Menz) could be associated with adaptation to different eco-climates and face different selection pressure, which may have shaped their genomes in different manner. Likewise, The close association observed among the three long fat-tailed sheep populations (Horro, Arsi-Bale, and Adilo) could be due to they are predominantly maintained by the same ethnic group or community, have same tail phenotype, and inhabit similar eco-climates (highland) and face common selective pressures, which may have shaped their genomes in a similar manner. The chances of animal exchange are greater within the same ethnic group or community than between any two different ethnic groups or communities (Gizaw et al. 2007).

The distant association observed between the short fat-tailed sheep, Menz, and the fat-rumped sheep, Black Head Somali, could be due to selection for ecological adaptation, geographical isolation and differences in tail phenotype (Gizaw et al. 2008; Edea et al. 2017). This finding is not in line with the report of Ahbara et al. (2019). The author indicated that the short fat-tailed, Molale-Menz, clustered together with the Ethiopian fat-rumped sheep which could be close proximity of sampling sites for the two different sheep populations since they are distinct in tail phenotype, adapted to different eco-climates, and maintained by different ethnic group or community which may have shaped their genomes differently (Gizaw et al. 2008).

The close association between Ethiopian fat-rumped sheep, BHS, and the Kenyan fat-tailed sheep, Red Maasai, could be because of the two sheep populations are reared under mobile pastoral and agro-pastoral systems and there is a high chance of exchanging animal across the border (Wilson 2011). They are also adapted to similar ecological environments (lowland) and face common selective pressures (Wilson 2011). The close clustering of Ethiopian fat-rump sheep (BHS) and Kenyan fat-tailed sheep (Red Maasai) suggests the dependent introduction and dispersion histories of Africa fat-tailed and fat-rumped sheep into the continent. This finding is agree with previous report that indigenous African sheep genetic resources have been classified into two main groups with a largely non-overlapping distribution: thin-tailed and fat-tailed (including fat-rump) sheep (Wilson 1991) but not in line with the report of Ryder (1984) which indicated that the fat-tailed sheep were introduced into Africa during the third wave of migration following thin-tailed hair sheep and thin-tailed wool sheep, fat-rumped sheep much later.

The close association of the two phenotypically different (variation in tail type) North African sheep populations could be due to gene flow between the two populations since they have close geographical proximity. Previous studies revealed that north Africa is mostly populated by fat-tailed sheep (Muigai and Hanotte 2013), but our STRUCTURE analysis indicated there is high signatures of admixture in the genomes of north Africa populations as compared to their east and South African populations and they shared 22–26% its genome with Middle East thin-tailed sheep (Iranian Afshari). The influence of Middle Eastern thin-tailed sheep detected in north Africa sheep can be explained by the historical introduction of sheep into Africa and their dispersion across the continent through the Nile Valley; for instance, thin-tailed sheep spread into the Western Sahara via Northern Africa (Muigai and Hanotte 2013), which may have left its genomic footprint in the current north Africa sheep populations.

The close clustering of east Africa sheep populations and distinct separation from their north counterparts was well demonstrated by our phylogenetic and PCA analyses (Figs. 3, 2a, respectively). This result coincides with the evidence that fat-tailed sheep were introduced into Africa via two independent routes: one via the north-east Africa and the Mediterranean Sea coastline, and the other via the Horn of Africa crossing through the strait of Babel-Mandeb (Ryder 1984).

The distinct clustering of Asian thin-tailed sheep from fat-tailed east and South Africa as well as from fat-rumped east Africa (Ethiopia) sheep suggests its independent introduction into the continent. It is in line with the distinct histories and non-overlapping geographic distributions of the African thin-tailed with fat-tailed and fat-rumped sheep (Hanotte et al. 2002; Muigai 2003b), and support the predominance of fat-tailed sheep in the east and South parts of Africa (Muigai and Hanotte 2013). Moreover, analyses of autosomal markers and the Y chromosome have revealed the distinct evolutionary histories of thin- and fat-tailed African sheep populations (Aswani 2007).

## Conclusions

Our genome-wide SNP analyses revealed that there is clear signature of admixture among Ethiopian sheep populations which could be accounted by some level of current admixture which results in low variation among the sheep populations but large within population variation. The principal component (PCA) and population structure analyses of Ethiopian sheep revealed four distinct genetic cluster groups according to their tail phenotype and geographical distribution. The short fat-tailed sheep populations did not represent one genetic cluster group which require further investigation. Our population structure analyses of Ethiopian sheep population demonstrated a clear pattern of the tail morphology and their phylogeography. Further investigation is required on morphometric basis of tail morphology variation in indigenous Ethiopian sheep populations to confirm the genetic basis of tail morphology variation investigated (Ahbara et al. 2019). Principal component and population structure analyses of global sheep population suggests the independent introduction of thin-tailed sheep into the continent but the dependent introduction and dispersion histories of fat-tailed and fat-rumped sheep. Principal component and phylogenetic analysis of global sheep population results coincide with the evidence that fat-tailed sheep were introduced into Africa via two independent routes: Horn of Africa, via the strait of Bab-el-Mandeb and Northern Africa, via the Isthmus of Suez from the Middle East (Ryder 1984).

## Electronic supplementary material

Below is the link to the electronic supplementary material.

Supplementary file1 (DOCX 105 kb)
