# Supplementary material for: Genome-wide insights of Ethiopian indigenous sheep populations reveal the population structure related to tail morphology and phylogeography
Source: Genes Genomics. 2020 Aug 16;42(10):1169–78. doi: 10.1007/s13258-020-00984-y (PMC7497517; doi:10.1007/s13258-020-00984-y)
Supplement: Supplementary file 1 — Supplementary file1 (DOCX 105 kb) [file 13258_2020_984_MOESM1_ESM.docx]

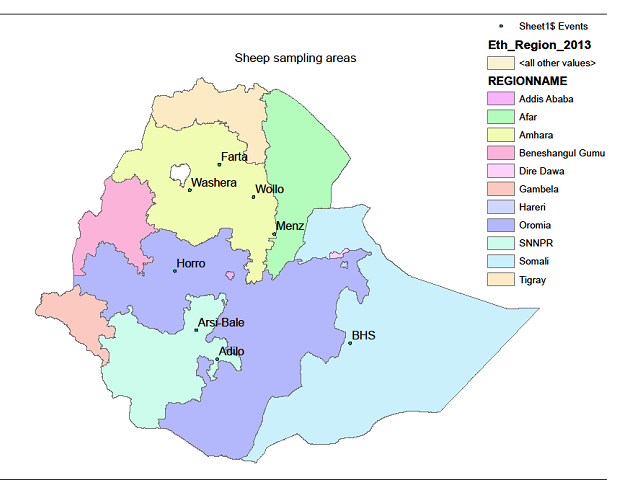


**Supplementary Fig. 1** Study sheep populations and their geographical locations

**Supplementary Table S1** Diversity indices for each of the fourteen sheep populations analyzed

| Population | N | *F* | *H*_O_ ± SD | *H*_E_ ± SD |
| --- | --- | --- | --- | --- |
| Horro | 30 | 0.009 | 0.342±0.16 | 0.352±0.14 |
| Wollo | 28 | 0.025 | 0.351±0.15 | 0.360±0.14 |
| Farta | 26 | 0.011 | 0.357±0.15 | 0.361±0.14 |
| Washera | 31 | 0.015 | 0.349±0.15 | 0.355±0.14 |
| Menz | 12 | -0.018 | 0.374±0.18 | 0.378±0.14 |
| Blackhead Somali | 13 | 0.012 | 0.356±0.18 | 0.363±0.14 |
| Arsi-Bale | 8 | 0.028 | 0.366±0.19 | 0.379±0.14 |
| Adilo | 11 | 0.041 | 0.353±0.18 | 0.368±0.14 |
| Red Maasai | 20 | -0.006 | 0.360±0.16 | 0.358±0.14 |
| Moroccan sheep | 21 | 0.056 | 0.379±0.14 | 0.401±0.11 |
| Egyptian Barki | 12 | 0.046 | 0.377±0.16 | 0.396±0.13 |
| Iranian Afshari | 20 | -0.018 | 0.383±0.16 | 0.376±0.13 |
| Namaqua Afrikaner | 12 | -0.139 | 0.411±0.20 | 0.363±0.15 |
| Indian Garole | 20 | 0.016 | 0.349±0.17 | 0.359±0.15 |

N: Number of genotyped individuals; *H*_O_: Observed heterozygosity; *H*_E_: Expected heterozygosity; SD: Standard deviation; *F*: Inbreeding coefficient level

**Supplementary Table S2** Proportions of the genetic admixture backgrounds in each fourteen sheep population (%)

| **Population** | **Cluster 1** | **Cluster 2** | **Cluster 3** | **Cluster 4** | **Cluster 5** | **Cluster 6** | **Cluster 7** | **Cluster 8** |
| --- | --- | --- | --- | --- | --- | --- | --- | --- |
| Washera | 0.008 | 0.001 | 0.012 | 0.360 | 0.001 | 0.123 | 0.496 | 0 |
| Wollo | 0.010 | 0.001 | 0.025 | 0.196 | 0.001 | 0.567 | 0.202 | 0.001 |
| Farta | 0.021 | 0.001 | 0.036 | 0.220 | 0.001 | 0.512 | 0.209 | 0.001 |
| Menz | 0.018 | 0.003 | 0.026 | 0.089 | 0.001 | 0.698 | 0.166 | 0 |
| Horro | 0.008 | 0.001 | 0.028 | 0.910 | 0.001 | 0.020 | 0.034 | 0 |
| Arsi-Bale | 0.007 | 0.003 | 0.280 | 0.618 | 0.001 | 0.068 | 0.024 | 0 |
| Adilo | 0.006 | 0.001 | 0.157 | 0.818 | 0.001 | 0.011 | 0.007 | 0 |
| BHS | 0.005 | 0.001 | 0.737 | 0.249 | 0.003 | 0.002 | 0.004 | 0 |
| Red Maasai | 0.025 | 0.004 | 0.698 | 0.239 | 0.010 | 0.004 | 0.001 | 0.020 |
| Iranian Afshari | 0.844 | 0.126 | 0.009 | 0.003 | 0.004 | 0.006 | 0.001 | 0.009 |
| Indian­ Garole | 0.037 | 0.947 | 0 | 0.001 | 0.001 | 0.003 | 0 | 0.013 |
| Namaqua Afrikaner | 0.003 | 0.001 | 0.003 | 0.001 | 0.999 | 0 | 0 | 0 |
| Egyptian Barki | 0.263 | 0.114 | 0.001 | 0.001 | 0.001 | 0.023 | 0.003 | 0.594 |
| Morrocan Sheep | 0.221 | 0.102 | 0.001 | 0.003 | 0.004 | 0.005 | 0.001 | 0.666 |

BHS= Blackhead Somali

**Supplementary Table S3** Proportions of the genetic admixture backgrounds in each eight sheep population (%)

| **Population** | **Cluster A** | **Cluster B** | **Cluster C** | **Cluster D** |
| --- | --- | --- | --- | --- |
| Washera | 0.243 | 0.553 | 0.204 | 0.001 |
| Wollo | 0.871 | 0.070 | 0.056 | 0.002 |
| Farta | 0.815 | 0.089 | 0.095 | 0.002 |
| Menz | 0.996 | 0.001 | 0.001 | 0.003 |
| Horro | 0.054 | 0.055 | 0.890 | 0.002 |
| Arsi-Bale | 0.139 | 0.031 | 0.614 | 0.217 |
| Adilo | 0.023 | 0.012 | 0.865 | 0.100 |
| BHS | 0.154 | 0.009 | 0.249 | 0.588 |

BHS= Blackhead Somali
